# Association of axillary node status with clinicopathological characteristics and expression of EZH2 and CD44 in primary breast ductal carcinoma

**DOI:** 10.12669/pjms.36.7.2954

**Published:** 2020

**Authors:** Miodrag Djordjevic, Aleksandar Karanikolic, Ljubinka Velickovic, Maja Milentijevic

**Affiliations:** 1 Miodrag Djordjevic, Clinic of Endocrine Surgery, Nis, Serbia. Medical Faculty, University Nis, Serbia; 2 Aleksandar Karanikolic, Clinic of Endocrine Surgery, Nis, Serbia. Medical Faculty, University Nis, Serbia; 3 Ljubinka Velickovic, Institute of Pathology, Nis, Serbia. Medical Faculty, University Nis, Serbia; 4 Maja Milentijevic, Institute of Pathology, Nis, Serbia. Medical Faculty, University Nis, Serbia

**Keywords:** Axillary node status, pN, EZH2, CD44, Breast cancer

## Abstract

**Objective:**

In order to enhance the prognostic benefit of new molecular markers, the aim of this study was to identify possible association of axillary lymph node (ALN) status and pN with clinicopathological characteristics and expression of EZH2 and CD44 in invasive ductal carcinoma (IDC) of the breast.

**Methods:**

The investigation included 106 patients with IDC who had undergone radical mastectomy at the Clinic of Endocrine Surgery in Nis. Clinicopathologic parameters and immunohistochemical expression of EZH2 and CD44 in primary IDC were investigated in relation to ALN status and pN.

**Results:**

Our univariate analysis established that T3-T4 stage, high EZH2, and high EZH2 with ER- were associated with ALN metastasis (p=0.014; 0.003; 0.013). Decreased probability for ALN involvement was found with T1 stage, and low EZH2 with ER+ (p=0.032; 0.022). Multivariant analysis established that high EZH2 in cancer cells was associated with high risk for ALN metastases (p=0.004); T1 tumors were associated with low risk (p=0.037). Higher pN was associated with high EZH2, high EZH2 with ER-, as well as an advanced clinical and disease stage (p=0.006; 0.001; p=0.002, 0.001). Lower pN was associated with ER+, and ER+ with low EZH2 (p= 0.004; 0.012). CD44 was not associated with ALN involvement, nor with pN.

**Conclusions:**

This study revealed association of EZH2 with ALN metastases, where disease stage and expression profiles of EZH2 and ER may have affected regional pN.

## INTRODUCTION

Breast cancer (BC) is the most diagnosed type of cancer in women throughout the world, and it has the second highest mortality rate in this group, after lung cancer.^1^-^3^ Invasive carcinoma of no special type (ductal NOS) is the most common histologic type of invasive BC, comprising 75% to 80% of newly diagnosed invasive tumors.^2^ Earlier diagnosis and improved treatment modalities of BC in the last several decades have led to the 5-year survival rates of BC patients to be raised to about 85%. Nevertheless, local and distant metastases still tend to occur and contribute to a poor prognosis of the disease.^4^ Presence of axillary lymph node (ALN) metastases is the most important prognostic factor for disease free and overall survival in the absence of distant metastasis and it is important for determining treatment.^5^

Expression profiles of hormone receptors (ER, PR), and Her-2/neu are closely associated with BC, being therefore used for prognosis and therapeutic response predictions.^4^ Some studies have shown that along with these parameters, Enhancer of Zeste Homolog 2 (EZH2) and CD44, molecular markers with cancer stem characteristics, have an important role in the prognosis of BC.^6^ EZH2 is the catalytic subunit of Polycomb repressive complex 2 (PRC2), which is a highly conserved histone methyltransferase that targets lysine-27 of histone H3. Studies on human tumors show that EZH2 is frequently over-expressed in a wide variety of cancerous tissue types, including BC, where is consistently associated with poor prognosis and aggressive behavior, including invasiveness and metastatic potential.^7^-^9^ EZH2 promotes BC progression by transcriptional repression of tumor suppressors and by maintaining the cells in a stem cells state.^7^ CD44 is a well-known marker with a role in promoting tumorigenesis of BC, including transformation, growth, cell invasion and motility, and is a marker of BC stem cells.^7^ In order to enhance the prognostic benefit of standard and new molecular markers on the occurrence of metastases in BC, the expression of EZH2 and CD44 was examined.

In order to enhance the prognostic benefit of new molecular markers, the aim of this study was to identify possible association of axillary lymph node (ALN) status and pN with clinicopathological characteristics and expression of EZH2 and CD44 in invasive ductal carcinoma (IDC) of the breast.

## METHODS

This study included one hundred six patients with invasive breast ductal carcinoma (IDC) who had undergone a radical mastectomy at Clinic of Endocrine Surgery, Clinical Center Nis, Serbia, between January 2016 and December 2019. It excluded the patients who received neoadjuvant therapy before surgery. Clinicopathologic parameters - patient age, pT (T1, T2, T3-T4), ALN metastasis (yes or no), pN (N0, N1, N2-3), clinical stage, grade of tumor (1,2,3), Nottingham score, ER/HER-2 status (negative, positive), Ki-67 index (low <20%, high ≥20%), and expression of EZH2 and CD44 were included in this study.

We performed immunohistochemistry on thin sections (4 μm) of tissue slides to examine the expression of EZH2 (clone ab84989, Abcam, 1/250 dilution), and CD44 (Dako, 1:50 dilution). The sections were cut, dried, deparaffinized, rehydrated, heat-pretreated using retrieval solution and stained with antibodies following standard procedures. Visible brown nuclear staining, predominantly moderate, was considered to indicate positive staining for EZH2, and membranous for CD44. The staining intensity was scored from 0 to 3 (no staining, weak, moderate, strong). The proportions of stained positive tumor cells were classified as 1 (0–25%), 2 (26–50%), 3 (51–75%) and 4 (76–100%). Multiplication for intensity and proportion was utilized to represent the protein levels of EZH2 and CD44. Based on the mean score for the entire study group, EZH2 expression in tumors was categorized into low and high expression groups. CD44 expression was scored as low, if ≤50% of cells were stained; and high, if >51% of cells were stained.^8^-^10^

Pearson chi-square test was used to assess the differences in distribution of investigated parameters among the patients with negative and positive ALN involvement and pN.The relationship between each investigated parameters and the risk of ALN metastasis was assessed using univariate logistic regression. Odds ratios (ORs) and their corresponding 95% confidence intervals (95% CIs) were calculated. We used backward LR method, which involves starting with all examined variables, to select variables that should be included in final multivariate regression model.

The tests were all two-sided and analyzed using the SPSS software package, version 18.0 (SPSS Inc., Chicago, IL, USA). The value of P < 0.05 was considered statistically significant.

## RESULTS

During the study period, 106 women underwent radical mastectomy because of IDC, and they did not have distant disease. Most of the studied patients (89.6%) were over 45 years of age. ALN metastases were detected in 60.4% women. Investigation of relationships between ALN status and investigated parameters showed that ALN metastases were closely linked to tumor stage (p=0.012), clinical stage (p=0.019) and expression of EZH2 (p=0.003). Low expression of CD44 was detected in 67% patients, and significant association with ALN status and pN was not detected (Table-I). In the univariate logistic regression analysis, T3-T4 tumor stage (OR=5.087; 95%CI: 1.394-18.654; p=0.014), clinical stage III (OR=5.018; 95%CI: 1.941-17.156; p=0.018), high EZH2 (OR=3.480; 95%CI: 1.526-7.939; p=0.003), and high EZH2 with negative ER status (OR=4.318; 95%CI: 1.356-13.746; p=0.013) were found to increase the probability for ALN involvement, while T1 tumor stage (OR=0.404; 95%CI: 0.176-0.926;p=0.032), clinical stage I (OR=0.420; 95%CI: 0.206-0.905; p=0.039) and low EZH2 with positive ER status (OR=0.327; 95%CI: 0.126-0.849; p=0.022) were associated with decreased probability for ALN involvement (Table-II). In the multivariate logistic regression model, T1 tumor stage (OR=0.393; 95%CI: 0.163-0.946; p=0.037) and high EZH2 (OR=3.479; 95%CI: 1.491-8.116; p=0.004) remained significantly associated with ALN status (Table-II).

**Table-I:** Association of lymph node involvement with clinicopathological characteristics and expression ofEZH2 and CD44 in primary IDC.

Parameter	No. of patients	Lymph nodes involvement		P Value

		Negative	Positive	
*Age, years*				
≤ 45 godina	11 (10.4%)	3 (7.1%)	8 (12.5%)	0.376
≤ 45 godina	95 (89.6%)	39 (92.9%)	56 (87.5%)	
*Grade*					
G1	6 (5.7%)	3 (7.1%)	3 (4.7%)	0.867
G2	64 (60.4%)	25 (59.5%)	39 (60.9%)	
G3	36 (34%)	14 (33.3%)	22 (34.4%)	
Nottingham score	6.85±1.18	6.93±1.02	6.81±1.30	0.626
*Pathologic stage, no.(%)*				
T1	35 (33%)	19 (45.2%)	16 (25%)	0.012
T2	50 (47.2%)	20 (47.6%)	30 (46.9%)	
T3 - T4	21 (19.8%)	3 (7.1%)	18 (28.1%)	
*pN, no.(%)*					
N0	42 (39.6%)	42 (100%)	0 (0%)		
N1	36 (34%)	0 (0%)	36 (56.3%)	0.000
N2-N3	28 (26.4%)	0 (0%)	28 (43.7%)	
*Clinical stage, no.(%)*				
I	24(21.6%)	19(45.2%)	5(7.8%)	
II	59(55.7%)	21(50.1%)	38(59.4%)	0.019
III	23(22.7%)	2(4.7%)	21(32.8%)	
*ER status*				
Negative, no.(%)	55 (51.9%)	19 (45.2%)	36 (56.3%)	0.267
Positive, no.(%)	51 (48.1%)	23 (54.8%)	28 (43.7%)	
*HER-2/neu status*					
Negative, no.(%)	76 (71.7%)	31 (73.8%)	45 (70.3%)	0.696
Positive, no.(%)	30 (28.3%)	11 (26.2%)	19 (29.7%)	
*Ki-67 index*					
Low (<20)	35 (33%)	11 (26.2%)	24 (37.5%)		
High (≥20)	71 (67%)	31 (73.8%)	40 (62.5%)	0.672
*CD44 no.(%)*				
Low	71 (67%)	30 (71.4%)	41 (64.1%)	0.430
High	35 (33%)	12 (28.6%)	23 (35.9%)	
*EZH2 no.(%)*					
Low	54 (50.9%)	29 (69%)	25 (39.1%)	0.003
High	52 (49.1%)	13 (31%)	39 (60.9%)	

**Table-II:** Logistic regression analysis of clinicopathological characteristics, expression of EZH2 and CD44 and lymph node involvementinprimary IDC.

Parameter	OR	95% C.I.		P value

		Lower	Upper	
*Univariate analysis*				
Age ≤ 45 years	0.538	0.134	2.158	0.382
G1	0.639	0.123	3.329	0.595
G2	1.061	0.479	2.349	0.884
G3	1.048	0.460	2.386	0.912
Stage I	0.420	0.206	0.905	0.039
Stage II	0.943	0.465	2.012	0.940
Stage III	5.018	1.941	17.156	0.018
Nottinghem score	0.920	0.661	1.281	0.622
T1	0.404	0.176	0.926	0.032
T2	0.971	0.445	2.116	0.940
T3 - T4	5.087	1.394	18.564	0.014
ER status positive	0.643	0.294	1.406	0.268
HER-2/neu status positive	1.190	0.497	2.846	0.696
High Ki-67 index	0.591	0.252	1.389	0.228
High EZH2	3.480	1.526	7.939	0.003
High CD44	1.402	0.604	3.255	0.431
High EZH2 and High CD44	1.057	0.321	3.482	0.927
Low EZH2 and Low CD44	0.603	0.275	1.321	0.206
High EZH2 and Low CD44	2.442	0.931	6.408	0.070
Low EZH2 and High CD44	0.714	0.238	2.143	0.548
High EZH2 and ER positive	1.548	0.622	3.852	0.347
Low EZH2 and ER negative	0.600	0.257	1.400	0.238
High EZH2 andER negative	4.318	1.356	13.746	0.013
Low EZH2 and ER positive	0.327	0.126	0.849	0.022
*Multivariate analysis*				
T1	0.393	0.163	0.946	0.037
High EZH2	3.479	1.491	8.116	0.004

The cancer spread to 1-3, or ≥4 axillary lymph nodes in 34%, ie. 26.4% patients. The higher pN was closely linked to tumors stage (p<0.001), clinical stage (p=0.002), high EZH2 (p=0.006) and high EZH2 with negative ER status (p<0.001). Lower pN was associated with positive ER status (p=0.004), as well aslow EZH2 with positive ER status (p=0.012) (Table-III).

**Table-III:** Association of pN with clinicopathological characteristics and expression ofEZH2 and CD44 in primary IDC.

Parameter	pN			P Value

	N0	N1	N2 - N3	
*Age, years*				
≤ 45 godina	3 (7.1%)	5 (13.9%)	3 (10.7%)	0.621
≤ 45 godina	39 (92.9%)	31 (86.1%)	25 (89.3%)	
*Grade*					
G1	3 (7.1%)	3 (8.3%)	0 (0.0%)	0.053
G2	25 (59.5%)	26 (72.2%)	13(46.4%)	
G3	14 (33.3%)	7 (19.4%)	15 (53.6%)	
Nottingham score	6.93±1.02	6.42±1.18	7.32±1.28	0.160
*Pathologic stage, no.(%)*				
T1	19 (45.2%)	11 (30.6%)	5 (17.9%)	0.000
T2	20 (47.6%)	23 (63.9%)	7 (25.0%)	
T3-4	3 (7.1%)	2 (5.5%)	16 (57.1%)	
*Clinical stage, no.(%)*					
I	19(45.2%)	5(10.6%)	0 (0.0%)		
II	21(50.1%)	38(80.9%)	0 (0.0%)	0.002
III	2(4.7%)	4(8.5%)	17(100%)	
*ER status*				
Negative, no.(%)	19 (45.2%)	14 (38.9%)	22 (78.6%)	0.004
Positive, no.(%)	23 (54.8%)	22 (61.1%)	6 (21.4%)	
*HER-2/neu status*					
Negative, no.(%)	31 (73.8%)	27 (75.0%)	18 (64.3%)	0.593
Positive, no.(%)	11 (26.2%)	9 (25.0%)	10 (35.7%)	
*Ki-67 index*					
Low (<20)	11 (26.2%)	19 (52.8%)	5 (17.9%)	0.060
High (≥20)	31 (73.8%)	17 (47.2%)	23 (82.1%)	
*CD44 no.(%)*					
Low	30 (71.4%)	23 (63.9%)	18 (64.3%)	0.732
High	12 (28.6%)	13 (36.1%)	10 (35.7%)	
*EZH2 no.(%)*					
Low	29 (69.0%)	16 (44.4%)	9 (32.1%)	0.006
High	13 (31.0%)	20 (55.6%)	19 (67.9%)	
High EZH2 and High CD44	5 (11.9%)	2 (5.6%)	6 (21.4%)	0.158
Low EZH2 and Low CD44	23 (54.8%)	17 (47.2%)	10 (35.7%)	0.294
High EZH2 and Low CD44	7 (16.7%)	13 (36.1%)	8 (28.6%)	0.145
Low EZH2 and High CD44	7 (16.7%)	4 (11.1%)	4 (14.3%)	0.782
High EZH2 and ER positive	9 (21.4%)	14 (38.9%)	5 (17.9%)	0.107
Low EZH2 and ER negative	15 (35.7%)	8 (22.2%)	8 (28.6%)	0.425
High EZH2 and ERnegative	4 (9.5%)	6 (16.7%)	14 (50.0%)	0.000
Low EZH2 and ER positive	14 (33.3%)	8 (22.2%)	1 (3.6%)	0.012

## DISCUSSION

ALN status remains one of the fundamental prognostic factors in BC and the TNM classification system remains the gold standard for staging the disease. ALN status has been used for guiding adjuvant, local or systemic treatment decisions.^5^,^11^ Wasuthit et al. suggested a combination of clinical, radiologic, and pathologic characteristics for the prediction of ALN involvement and selection of BC patients for full ALN dissection.^12^

EZH2 is essential in a number of important cellular processes, such as embryonic and adult stem cell maintenance and tumor progression.^13^ Depletion of EZH2 in BC cells significantly increased expression of the top altered genes, decreased proliferation, and improved cell adhesion, indicating a critical role played by EZH2 in determining the cancer phenotype.^14^

Having in the mind that a key event in the progression of BC is the development of lymph node metastases, it is very important to determine prognostic benefit of standard and new molecular markers (ER, HER2, EZH2, CD44) on the occurrence of metastatic disease. This study detected that patients with advanced tumor stage, high EZH2, and high EZH2 with negative ER status had a high risk for ALN metastases in the univariate analysis (p=0.014; 0.003; 0.013). Low risk for ALN involvement was detected in patients with T1 stage, and tumors with positive ER status and low EZH2 (p=0.032; 0.022). Multivariate analysis detected that ALN metastases were significantly associated with high expression of EZH2 in cancer cells (p=0.004), and T1 stage had a low risk for ALN involvement (p=0.037).

Our results are consistent with the studies of some other authors who detected that overexpression *of EZH2* was associated with larger tumor size, advanced disease, and significantly worse disease free and overall survival than those with tumors expressing low EZH2, but added that overexpression *of EZH2* was associated with negative ER status.^15^,^16^ Reijm et al. showed that high *EZH2* expression was associated with the lymph node status only in univariate analysis. They found significant positive associations with the number of lymph nodes involved, histologic grade and HER2 status.^17^

Biological evidence has shown that overexpression of EZH2 induces type 1 histone deacetylation (HDAC) enzymatic activity in breast epithelial cells. Furthermore, the HDAC activity induced by EZH2 may explain the strong association between EZH2 protein expression and negative ER, showing that EZH2 may transcriptionally repress ER.^18^,^19^

Recent findings of Yomtoubian et al. showed that specific pharmacological or genetic inhibition of EZH2 catalytic activity impairs distant metastasis in triple negative breast cancer patients. EZH2 inhibition differentiates EZH2 high basal cells to a luminal-like phenotype by derepressing GATA3 and renders them sensitive to endocrine therapy.^20^ Polycomb inhibitors, especially those directed against the PRC2 catalytic subunit EZH2 have shown responses in preclinical studies of cancer therapy.^9^

The number of lymph nodes involved has emerged as a prognostic factor in determining BC prognosis. Patients with four or more positive lymph nodes have a worse prognosis compared to those having three or fewer histologically positive lymph nodes.^21^ This study identified that higher pN was associated with some parameters which influenced ALN involvement, i.e. with high EZH2, high EZH2 with negative ER status, advanced clinical stage and advanced tumors stage (p=0.006; 0.001; p=0.002; 0.001). Patients with pN2-N3 had high EZH2 in 67.9%, and high EZH2 with negative ER status was seen in 50%. However, lower pN can be expected in women with positive ER status separately (p=0.004) or together with low EZH2 expression (p=0.012). Despite the strong association of EZH2 with ALN status, our study has some limitations having in the mind that it was done in one center.

Some studies indicate that multifunctional cell adhesion molecules CD44 are potential markers of tumour progression, and may favor distant metastasis.^22^ This study investigated the expression of CD44 to clarify its impact on ALN status and pN. Low CD44 was present in 67% of IDC. A high EZH2 expression with CD44 reduction in cancer cells had a certain impact on ALN involvement (OR 2.442, 95% CI 0.931–6.408, p=0.070), but a significant association was not detected.

## CONCLUSION

Despite some limitations, (the study has been conducted on the sample size from only one center) this study points the association of high EZH2 expression in IDBC with ALN metastases, where increasing pN was associated with more advanced tumor and clinical stage, high EZH2, and high EZH2 with negative ER status. Our study showed that EZH2 and ER can facilitate regional lymph node metastatic progression in women with BC.

### Authors’ Contribution

**MD** conceived, designed and did statistical analysis & editing of manuscript and is responsible for integrity of research.

**MD, AK, LjV** did data collection and manuscript writing.

**MM** did review and final approval of manuscript.
